# High-Sensitivity Gas-Phase Raman Spectroscopy for Time-Resolved In Situ Analysis of Isotope Scrambling over Platinum Nanocatalysts

**DOI:** 10.1021/acs.analchem.5c02840

**Published:** 2025-08-13

**Authors:** K. Koschnick, A. M. Ferris, B. Zhang, J. Lill, M. Stark, A. Weinmann, H. H. Limbach, T. Gutmann, D. Geyer, A. Dreizler

**Affiliations:** † Reactive Flows and Diagnostics, Department of Mechanical Engineering, Technical University of Darmstadt, Otto-Berndt-Straße 3, 64287 Darmstadt, Germany; ‡ Optical Diagnostics and Renewable Energies, Department of Mechanical and Plastics Engineering, University of Applied Sciences Darmstadt, Schöfferstraße 3, 64295 Darmstadt, Germany; § Department of Mechanical and Aerospace Engineering, 6740 Princeton University, Princeton, New Jersey 08544, United States; ∥ Eduard-Zintl-Institute for Inorganic and Physical Chemistry, Technical University of Darmstadt, Alarich-Weiss-Straße 4, 64287 Darmstadt, Germany; ⊥ Algorithms for Computer Vision, Imaging and Data Analysis, University of Applied Sciences Darmstadt, Schöfferstraße 3, 64295 Darmstadt, Germany; # Institute of Chemistry and Biochemistry, Freie Universität Berlin, Arminallee 22, 14195 Berlin Germany

## Abstract

In this study, we present a novel approach for time-resolved, in situ analysis of isotope scrambling reactions over platinum nanoparticle catalysts using high-sensitivity gas-phase Raman spectroscopy. A recently developed spectrometer setup enables detection limits in the hundreds of ppm, a dynamic range spanning four orders of magnitude in mole fraction, and a temporal resolution of one second. Experiments were performed by introducing D_2_ gas to an H_2_-activated Pt nanoparticle catalyst in a closed sample, resulting in the formation of gaseous HD and H_2_. The time-resolved gas-phase mole fraction profiles show HD as the dominant product and only minor formation of H_2_. This observation is consistent with a predominantly associative exchange mechanism, in which D_2_ reacts directly with surface-bound hydrogen to produce HD. A superimposed exchange involving trace water vapor was also observed, with stepwise conversion of H_2_O to HDO and D_2_O via surface-mediated reactions. Mole fractions were quantified using a spectral fitting routine based on simulated Raman spectra derived from literature polarizabilities and energy levels. The reaction quotient of the hydrogen isotopologues converged over time toward literature values of the equilibrium constant, and measurements at defined H_2_/D_2_ ratios confirmed relative accuracies better than 2%. This Raman-based quantification method enables simultaneous, in situ detection of all relevant species with high accuracy and is ideally suited for studying transient, catalytic processes.

## Graphical abstract

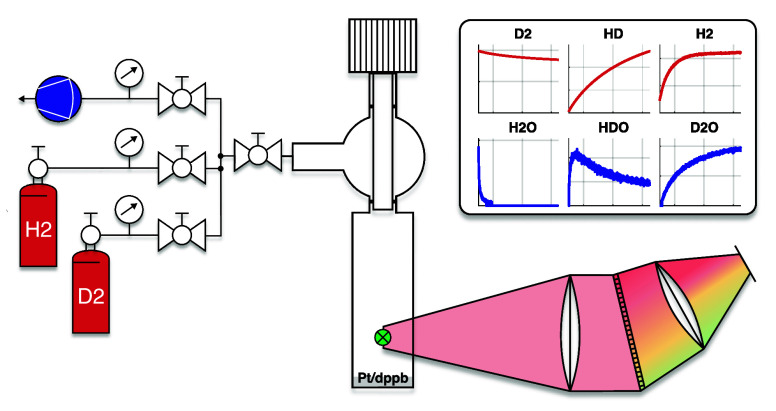

## Introduction

Raman spectroscopy in the gas phase is a highly specific and versatile diagnostic technique, capable of simultaneously quantifying multiple chemical species via their unique vibrational and rotational energy states.^1^ It enables both spatial and temporal resolution as well as real-time monitoring of gas-phase temperatures, making it invaluable in studying reactive flows such as flames.^1,2^ More recently, gas-phase Raman spectroscopy has been applied to the study of heterogeneous chemistry, including catalysis and electrochemistry.^3–5^ In prior work, we introduced a novel dual-track Raman spectrometer (DTRS) platform capable of capturing either both polarization orientations or two spectral resolutions simultaneously across two optical tracks, while maintaining high spatial and temporal resolution.^6^ This study applies the DTRS to investigate a transient hydrogen–deuterium exchange reaction over a nanoparticle catalyst, leveraging its time-resolved capabilities to probe reactions and kinetics under in situ conditions.

Nanoparticle catalysts, consisting of transition metals stabilized by organic ligands, are a promising catalyst system for C–H bond activation, e.g., for alkane hydrogenolysis, where the ligand can be used to fine-tune activity and selectivity.^7,8^ However, deeper understanding of the species adsorbed on the nanoparticle surface is required, particularly in terms of mobile hybrid species.^9^ It has been shown that these hybrids are exchangeable by deuterium.^9^ Therefore, hydrogen/deuterium (H/D) exchange reactions can be used to test the ability of the catalyst to activate C–H bonds.^10^ These isotope exchange reactions have been studied by Pery et al.^9^ and Limbach et al.^11^ with joint ^2^H solid-state nuclear magnetic resonance (NMR) spectroscopy and ^1^H NMR spectroscopy in the gas-phase for ruthenium nanoparticles. The measurements were performed at room temperature and at a pressure of 800 mbar in a closed NMR glass tube. Having a closed reactive sample allowed for studying the system under conditions where the number of hydrogen atoms in the gas phase is comparable to that on the surfaces of the metal nanoparticles.^11^ Rothermel et al.^7^ applied this approach to platinum-based catalyst systems up to 2 bar. The overall isotopic exchange observable in the gas phase is summarized by the reaction

1 $$H_{2}+D_{2}⇌2\mathrm{HD}$$

though this represents the net result of surface-mediated processes rather than a direct gas-phase reaction. Limbach et al.^11^ used gas-phase NMR to demonstrate that the dominant exchange mechanism on ruthenium nanoparticles is associative, as primarily HD was detected in the gas phase. According to Limbach et al., the associative pathway involves molecular adsorption of D_2_ on the catalyst surface, exchange with a surface-bound hydrogen, and associative desorption of HD. In contrast, the dissociative mechanism entails initial cleavage of D_2_, surface diffusion of atomic deuterium, and recombination with surface hydrogen.^11^ While both associative and dissociative mechanisms ultimately lead to equilibrium product distributions in this experiment, their transient behavior differs: an associative mechanism yields HD as the dominant early product, whereas a dissociative pathway would result in a more balanced mixture of H_2_, HD, and D_2_ even at early stages.^11^ A detailed discussion of the surface processes is provided by Limbach et al.^11^ In their work, quantifying both H_2_ and HD simultaneously via ^1^H NMR poses the challenge of superimposed signals that can only be discriminated by their differing line-width.^11^ This superposition has to be solved by line shape analysis, which suffers from background that interferes with the actual signal. The analysis is further complicated in cases, where one of the components H_2_ or HD is present in low mole fractions of less than 10%. Additionally, it is not possible to measure the D_2_ concentration simultaneously. Since H_2_ could not be confidently distinguished from the background, Limbach et al.^11^ assumed that there is no significant production of H_2_ in the reaction.

Studying H-D-isotope exchange in closed systems requires real-time, in situ diagnostics with high temporal resolution and species sensitivity. Extractive techniques, such as gas chromatography or mass spectrometry, typically suffer from low temporal resolution and necessitate a gaseous sample to be extracted from the reacting system, an intrusive event that is unsuitable for a study in which the volume of gas in the reacting system is small. Fourier-transform infrared spectroscopy (FTIR) cannot detect homonuclear diatomic molecules (H_2_, D_2_, N_2_, O_2_), which are central to this study. In contrast to ^1^H NMR and FTIR, gas-phase Raman spectroscopy is capable of detecting both homonuclear diatomic molecules and more complex species simultaneously. Rothermel et al.^7^ demonstrated Raman detection in H/D exchange but only at a qualitative level, with insufficient signal-to-noise for H_2_ detection at low concentrations. Schlösser et al.^12^ used a similar isotope scrambling reaction to calibrate their Raman system with subpercent precision. However, their setup targeted equilibrium mixtures and did not aim to resolve kinetics; water isotopologues were also intentionally excluded.

Achieving a low limit of detection (LOD) in gas-phase Raman spectroscopy remains challenging due to the inherently weak spontaneous Raman cross section. Fiber-enhanced systems, such as the one demonstrated by Knebel et al.,^13^ can achieve LODs ranging from subppm to several hundred ppm, depending on the target molecule and pressure. However, these systems typically require long integration times and extractive sampling. Cavity-enhanced techniques, as employed by Yang et al.,^14^ reach subppm levels, but are incompatible with commercial cuvettes used to contain samples and are sensitive to particle or dust contamination, limitations critical in a catalysis setups. Multipass systems as in Kim et al.^15^ face even greater geometrical challenges in closed sample configurations. Thus, although various Raman techniques offer high sensitivity, they are either intrusive or incompatible with closed sample systems. To our knowledge, no existing approach achieves detection limits in the hundreds of ppm for all major species simultaneously while maintaining one-second temporal resolution in a nonintrusive setup.

In this context, Raman spectroscopy using the DTRS as a unique tool opens a new pathway for the study of transient catalytic processes such as H-D exchange reactions in closed reacting systems. As shown in this work, its ability to simultaneously detect and temporally resolve all relevant gas-phase species at low LODs allows detailed kinetic analysis and insight into surface reaction mechanisms of metal nanoparticle catalysts.

## Experimental Section

### Raman Spectrometer Setup

The Raman setup used in this study was previously described in detail by Koschnick et al.^6^ The custom-designed transmission spectrometer, optimized for detecting Raman spectra in the visible range with excitation at 532 nm, is shown in Figure S1 of the Supporting Information. The system uses a 90° scattering geometry, in which the signal is collected perpendicular to the laser axis. Key optical elements include a combination of photographic lenses (Pentax and Zeiss), a long-pass filter (BLP01-532R-50, Semrock; cut-on at 542 nm) to suppress Rayleigh and anti-Stokes scattering, and an absorbing polarizer (ProFlux ABGS5C, Moxtek) to attenuate unpolarized background light by approximately 50%. A complete list of optical components is provided in Table S1 of the Supporting Information. For this study, the Raman signal was dispersed by a wide-range holographic transmission grating (G10-V01, Wasatch Photonics) with a spatial frequency of 1064 l/mm and a diffraction angle of 19.5° under Bragg conditions. This configuration yields a spectral range of approximately 515–4650 cm^–1^, enabling simultaneous detection of all relevant species in this study: H_2_, D_2_, HD, H_2_O, D_2_O, HDO, N_2_, and O_2_. The spectral resolution was approximately 19.5 cm^–1^ (full width at half-maximum) for this particular study. The Raman signal was focused onto a back-illuminated, thermo-electrically cooled charge-coupled device (CCD) (SOPHIA 2048b-152-VS-X, Teledyne Princeton Instruments; 2048 × 2048 pixels with 15 μm pitch).

The camera and leaf shutter were externally triggered, allowing adjustment of the interval between acquisitions when high temporal resolution was not required, while maintaining a constant exposure time and, consequently, a consistent signal level. As spatial resolution was not relevant for this study, the CCD was fully binned along the spatial axis, yielding an effective data format of one (super)­pixel spatially and 2048 pixels spectrally. This corresponds to an effective probe volume with a horizontal extent of approximately 6 mm and a diameter (or height) of about 0.1 mm.

The Raman signal was excited using a continuous wave (CW) fiber laser (GLR-100-532, IPG Photonics), frequency-doubled to 532 nm and vertically polarized. To avoid excessive laser irradiance that could damage the cuvette, the laser power was limited to 10 W (out of 100 W total possible output). The laser beam was focused at the center of the cuvette using a 500 mm lens.

### Isotope Exchange Experiment Setup

A cuvette made of fused silica (700/3/Q, Starna) was used as an optically accessible reactor for the H–D exchange reaction. The cuvette, shown in Figure 1, has four polished sides and a square base, measuring 12.5 mm × 12.5 mm externally and 10 mm × 10 mm internally. A stopcock mounted on top of the cuvette allowed the lower part to be sealed after filling. Together with a height of approximately 40 mm, this results in an internal volume of 4 cm^3^. A thermocouple was placed beneath the cuvette to monitor the catalyst temperature. The cuvette was filled with 20 mg of catalyst particles, which covered the bottom surface. The laser, and thus the probe volume, was positioned 10 mm above the catalyst bed to prevent potential interference from fine particles adhering to the cuvette walls near the bottom, which could scatter or absorb the high-power laser beam. To estimate the time scale of diffusive transport from the catalyst surface to the probe volume, we applied Fick’s second law of diffusion in one dimension. The characteristic diffusion time *t*_diff_ for a particle to travel a distance *L* is given by

2 $$t_{\mathrm{diff}}=\frac{L^{2}}{2D}$$

where *D* is the binary diffusion coefficient. Assuming *D* = 0.2 cm^2^ s^–1^ for H_2_ in D_2_ at 3 bar and room temperature (estimated using the Fuller correlation^16^) and *L* = 10 mm, we obtain *t*_diff_ ≈ 2.5 s. This is of the same order of magnitude as the temporal resolution of the experiment (1 s), suggesting that diffusion may influence the steepness of observed temporal gradients, particularly at the beginning of the reaction. However, as shown later, the investigated reaction kinetics are significantly slower. Therefore, any convolution of diagnostic temporal resolution and diffusive transport is not expected to affect the results, particularly beyond the initial phase of the experiment.

**Figure 1:**
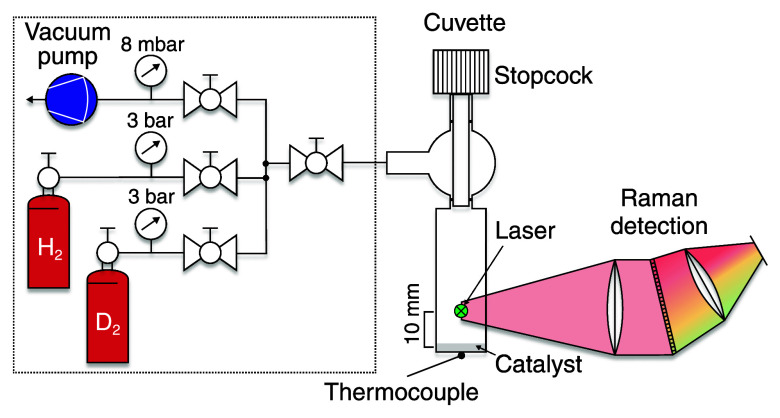
Schematic representation of the hardware for the isotope exchange experiment. The dashed box contains all the media supply. The cuvette is shown on the right side. The Raman detection is heavily simplified in the schematic.

The synthesis of the platinum nanoparticles stabilized with 1,4-bis­(diphenylphosphino)­butane (Pt/dppb nanoparticles) used as the catalyst in this study followed the metal–organic approach introduced by Chaudret et al.^17^ Details on the synthesis are given by Rothermel et al.^7^ It should be noted that the nanoparticles were stored in a glovebox under an argon atmosphere prior to use. Then 20 mg of the platinum (Pt) nanoparticles were filled into the cuvette, all valves were opened, and a membrane pump (PC 3001 Vario select, Vacuubrand) was used to evacuate the entire system, including the cuvette and connecting lines, down to approximately 8 mbar for 15 min. Following evacuation, the catalyst activation process was initiated by closing the valve to the pump and opening the H_2_ gas bottle, with the pressure regulator set to 3 bar. After 90 min of hydrogen exposure for activation, the system was evacuated again at 8 mbar for another 15 min. Before introducing deuterium gas into the system, the spectrometer recording was started, and the catalyst temperature was monitored via the thermocouple. The time of zero for the measurement was defined as the moment the D_2_ valve was fully opened.

Once the cuvette had been filled with D_2_ at 3 bar, the stopcock was closed to define the sealed reaction volume. The total measurement duration was 5500 s. As the reaction rate is highest at the beginning and gradually slows over time, the interval between consecutive 1 s exposures was progressively increased to optimize data acquisition and reduce unnecessary stress on the leaf shutter. At the beginning of the reaction, the time between exposures was kept at the CCD readout time of approximately 130 ms. After 1150 s, the interval was increased to one second, and after 3060 s, it was further increased to three seconds.

### Quantification Methodology

A calibration and data processing procedure was developed to derive time-resolved absolute mole fractions from the data recorded by the spectrometer’s CCD array. The method builds upon tools introduced in previous publications^6,18–20^ and, for this study, consisted of three calibration steps followed by a spectral fitting routine. The calibration steps were:

- *Spectral axis calibration*: Wavelengths were mapped to the spectral axis pixels using a third-order polynomial fit, based on the emission spectrum of a low-pressure neon lamp (Newport Photonics).
- *Spectral transmission efficiency*: As the spectrometer’s response varies across the spectral range, an intensity response function was determined. This was done using NIST *Standard Reference Material 2242a*, following the procedure developed by Schlösser et al.^21^ for 90° scattering configurations. The reference material was excited at 50 mW laser power with polarization set to equal horizontal and vertical shares, and a fused silica glass window with similar dimensions was placed in the beam path to simulate the effect of the cuvette walls.
- *Gas calibration*: The relative Raman cross sections of O_2_, H_2_, and H_2_O, using nitrogen as a reference gas, were recorded in an optically accessible flow channel preheated to 454 K to prevent water condensation on surfaces. The change of Raman cross section relative to the experiment conditions at room temperature was accounted for via ab initio calculations.^29^ The usage of these cross-section ratios in the spectral quantification process is described later in this section.

To evaluate the spectra of all relevant species simultaneously, an advanced spectral fitting routine was employed, building on our previous work.^6,19,20^ A conceptually related Bayesian formulation was previously applied by Bahr et al.^22^ in the context of rotational Raman thermometry. While Bayesian approaches to spectroscopic data analysis are well established,^23–25^ our method advances the state of the art by implementing an integrated framework for noise handling, background correction, and spectral analysis. This unified approach minimizes error propagation compared to conventional stepwise procedures. It enables high-precision multispecies fitting for up to 14 species, with potential for further extension. The background signal is modeled using a penalized spline, allowing for greater flexibility and accuracy than traditional low-order polynomial fits. Furthermore, more complex line shape models are employed to capture asymmetric features, further reducing model errors. In summary, the approach uses a Levenberg–Marquardt algorithm to solve a weighted least-squares optimization problem:

3 $$\begin{array}{ll} \underset{x}{\mathrm{argmin}}f\left(\mathrm{\nu ̃},x\right) & ={∥\frac{S_{E}\left(\mathrm{\nu ̃}\right)-S_{L}\left(\mathrm{\nu ̃},x\right)-S_{B}\left(\mathrm{\nu ̃},x\right)}{\sigma_{S_{E}\left(\mathrm{\nu ̃},x\right)}}∥}_{2}^{2}\\ & +\beta {∥\nabla^{2}S_{B}\left(\mathrm{\nu ̃},x\right)∥}_{2}^{2} \end{array}$$

Here, ν̃ denotes the Raman shift vector, and *S*_E_ is the experimentally measured signal. The synthetic signal *S*_L_ is a linear combination of individual species libraries, each described by a set of fitting parameters *x* = {ζ_*i*_,Δ*v*_*i*_,α,γ_*D*,*i*_,γ_0,*i*_,γ_2,*i*_,δ_*D*,*i*_,δ_0,*i*_,η_*i*_,ν_*VC*,*i*_}. These include the scaling factor ζ_*i*_ (elaborated on below), a shift parameter Δ*v*_*i*_, the background parameters α, and seven convolution kernel parameters (γ_*D*,*i*_, γ_0,*i*_, γ_2,*i*_, δ_*D*,*i*_, δ_0,*i*_, η_*i*_, ν_*VC*,*i*_) for the Hartmann–Tran line shape model, introduced by Ngo et al.,^26^ which represents the apparatus or transfer function. The noise on each pixel’s signal was estimated following McCreery^27^ as

4 $$\sigma_{S_{E}\left(\mathrm{\nu ̃},x\right)}=\sqrt{S_{L}\left(\mathrm{\nu ̃},x\right)+S_{B}\left(\mathrm{\nu ̃},x\right)+\sigma_{\mathrm{CCD}}^{2}\left(\mathrm{\nu ̃}\right)}$$

where *S*_L_ is the synthetic Raman signal, *S*_B_ the background signal, and σ_CCD_ includes the experimentally determined readout noise of the CCD as well as a small contribution from dark current at an exposure time of 1 s. The background signal *S*_B_ was modeled as a linear combination of basis splines, parametrized by α, as introduced by Eilers and Marx.^28^ The smoothness of the background fit was regularized by the parameter β, which penalizes the second derivative of the spline background. The species libraries consisted of quantum-mechanically simulated spectra that were convolved with the apparatus function. The simulation methodology is explained by Lill et al.^29^ Since the experiment in this study was performed at a steady temperature of 22 °C, it was not necessary to simultaneously optimize for the temperature in the fitting routine and the library spectra at the thermocouple temperature were used. An exemplary fitted experimental spectrum in the rovibrational region of the hydrogen isotopologues for a measurement 115 h after the reaction started averaged over 200 frames is shown in Figure 2.

**Figure 2:**
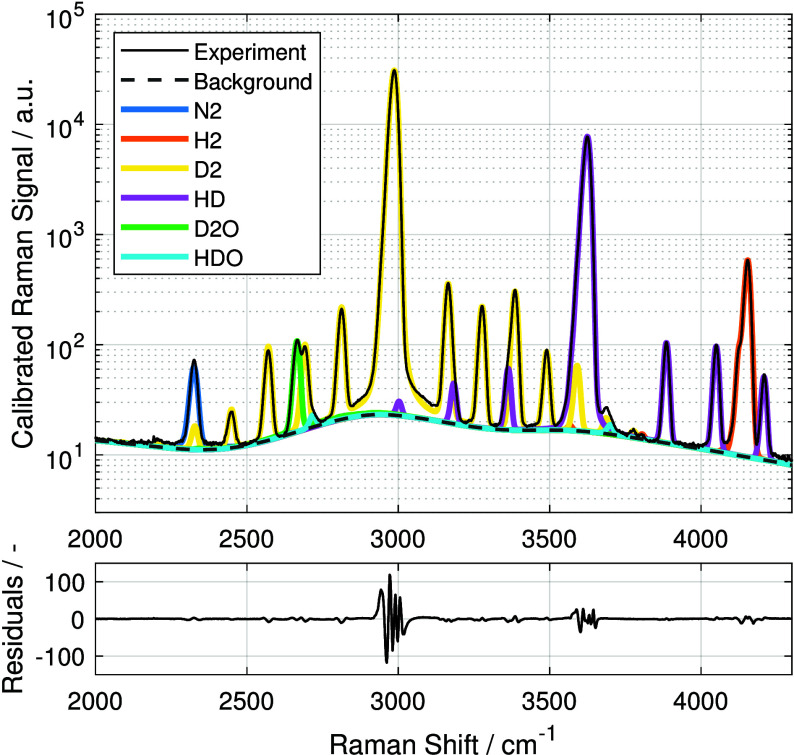
Fitted rovibrational Raman spectrum of 200 frames averaged at 115 h after the experiment started. Note that the ordinate is logarithmically scaled to make smaller transitions and species visible. The black thin line for the experimental data is almost perfectly resolved by the individual species libraries (colored) and the background, as shown in the residuals.

The primary objective of the fitting routine was to determine the linear scaling parameters ζ_*i*_ for each species. These parameters were then used to calculate species mole fractions via the molecule-specific and temperature-dependent Raman cross-section, κ_*i*_. Since it is generally difficult to calculate absolute Raman cross sections from quantum mechanical simulations, these values typically need to be obtained experimentally. To determine the relative Raman cross sections, calibration measurements were carried out using known mixtures of N_2_ and H_2_, N_2_ and O_2_, as well as N_2_ and H_2_O, in an optically accessible flow channel preheated to 454 K to prevent water condensation. For this case, N_2_ was dosed using a mass flow controller (Bronkhorst, El-Flow Prestige), while H_2_O was introduced via a syringe pump (Hitec Zang, SyrDos) and a small-scale vaporizer (ChemTherm, TV.1). The combined uncertainty of this setup is estimated to be within 2%. The H_2_/N_2_ calibration was performed using a certified premixed gas bottle (AirLiquide, stated accuracy 2% relative), and dry air was used for the O_2_/N_2_ calibration. The resulting relative cross sections κ_H_2__/κ_N_2__ and κ_H_2_O_/κ_N_2__ were then corrected for temperature differences to the isotope scrambling experiment using quantum-mechanical simulations described in ref 29.

For the rarer isotopologues of hydrogen and water, such calibrations are challenging and cost-intensive. Thus, the spectra and their relative cross sections were simulated using transitions and polarizabilities from literature. Raj et al.^30^ calculated polarizabilities ab initio for vibrational transitions of H_2_, D_2_, and HD as a means to calibrate the spectral transmission efficiency of their spectrometer. They expected a relative cross-section accuracy of 1–2% based on experimental data validation.^31^ Avila et al.^32^ validated their ab initio calculations of the water isotopologues with experimental data at known mixtures of H_2_O, D_2_O, and HDO, with an estimated error of less than 5%. Based on this knowledge, we calculated the relative cross-section, for instance, κ_HD_/κ_H_2__, from the simulation by Raj et al.,^31^ and then further related this to our reference molecule N_2_ using the experimentally derived ratio κ_H_2__/κ_N_2__. The result was a vector κ_*i*_/κ_N_2__ that contained the relative cross-section of any molecule of interest with respect to N_2_.

The individual mole fractions *x*_*i*_ were calculated based on established Raman quantification principles using relative cross sections.^27,33^ The specific formulation used here follows the implementation by Beumers et al.:^34^

5 $$x_{i}=\frac{\zeta_{i}}{\overset{n}{\underset{j=1}{\sum }}\zeta_{j}\cdot \frac{\kappa_{N_{2}}}{\kappa_{j}}}\cdot \frac{\kappa_{N_{2}}}{\kappa_{i}}$$

where *j* is the index for each species in the sum, and *n* is the total number of species. It should be noted that both the pure rotational response (*v* = 0) and the rovibrational response (*v* = 0 → 1) of H_2_, D_2_, and HD were captured in the experiment. As these spectral signatures span almost the full width of the sensor, combining them into a single library poses challenges for the fitting routine, since only one set of parameters for the spectral shift and the apparatus function is applied across a wide Raman shift range of more than 3500 cm^–1^. Small deviations in pixel-to-wavelength calibration or optical alignment of the spectrometer can make such a combined approach error-prone. Therefore, the libraries for pure rotational and rovibrational responses were separated and treated as individual species during fitting. However, only the rovibrational responses, exclusively shown in Figure 2, were used for mole fraction determination in eq 5, owing to their higher signal-to-noise ratio (SNR).

To quantify the uncertainty in the retrieved mole fractions, uncertainties in the fitted scaling parameters ζ_*i*_ and the relative Raman cross sections κ_*i*_ were propagated using Jacobian-based uncertainty propagation. Variances for ζ_*i*_ were extracted from the covariance matrix, as described by Bahr et al.,^22^ accounting for both measurement noise and the ill-posedness of the inverse problem. The uncertainty in each cross-section κ_*i*_ depended on its origin: for experimentally calibrated species, inverse-variance weighting was applied to all representations of the calibration coefficient to obtain the estimate with the lowest variance; for simulated species, literature-based uncertainty estimates were propagated through relative ratios (e.g., κ_HD_/κ_H_2__) using Jacobian-based uncertainty propagation. The resulting variances for both ζ_*i*_ and κ_*i*_ were then propagated through eq 5 to obtain the final error estimate on *x*_*i*_. These uncertainties (e.g., $\sigma_{x_{D_{2}}}$ ) are reported as absolute values alongside the mole fractions (see Data Availability) and range from approximately 30% relative uncertainty for D_2_O and H_2_O near their detection limit to 0.04% for D_2_ at the beginning of the experiment. It should be emphasized that these values primarily reflect the precision of the quantification method rather than its accuracy. Systematic errors, such as drifts during the experiment, drifts occurring between calibration and measurement, or inaccuracies in the spectral transmission efficiency calibration, are not captured in the statistical uncertainty. The accuracy is therefore assessed separately in the following section.

## Experimental Validation

To prove the accuracy of the data presented here an experimental validation approach was performed. Using two mass flow controllers (MFCs) (Bronkhorst, El-Flow Prestige), H_2_ and D_2_ were flowed through the empty cuvette with the top cap open, producing a homogeneous binary gas mixture at atmospheric pressure and room temperature. The mole fraction ratio was varied between measurements to assess the accuracy of the Raman data quantification at different mixture compositions. It was not feasible to perform this validation for all species of interest, as pure HD is difficult to isolate and maintain due to isotopic scrambling, and HDO is expensive and challenging to prepare in defined concentrations. While D_2_O is relatively inexpensive, its gas-phase dosing remains nontrivial. Therefore, the validation was limited to H_2_ and D_2_, for which gas mixtures could be prepared reliably. The resulting accuracy estimation is considered representative for the other species, given the shared spectral fitting and detection framework. The MFCs used for the H_2_-D_2_ experiment are specified with a relative uncertainty of 1% of the current value plus 1% of the full-scale value. This error was propagated to the mole fraction of hydrogen in a binary mixture using Gaussian error propagation. Figure 3 shows the results of this experiment. The set mixture is indicated by black squares and was varied from 29% D_2_/71% H_2_ to the inverse ratio.

**Figure 3:**
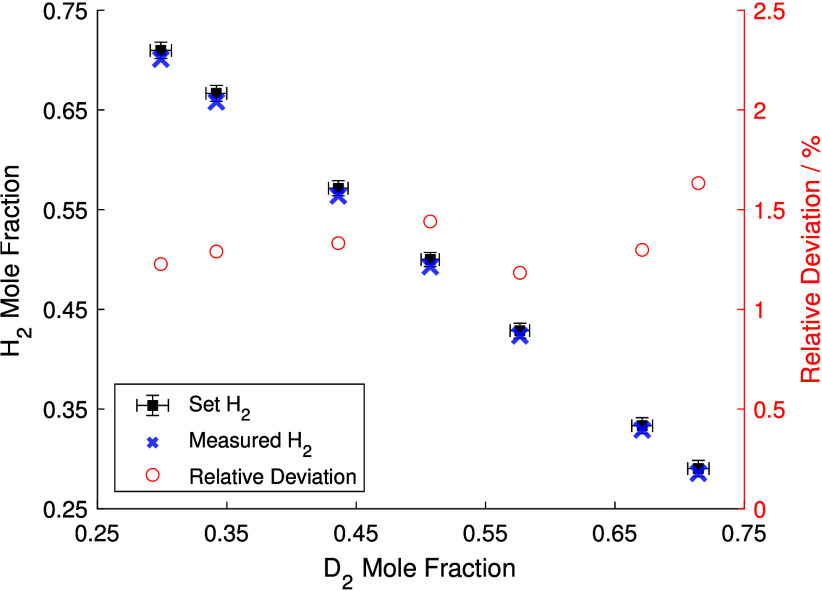
Validation of Raman quantification using controlled H_2_/D_2_ mixtures in an open cuvette. The set mixture ratios (black squares) were compared to the measured mole fractions of H_2_ (blue crosses). The relative deviation is plotted on the right axis.

The measured mixture fractions, shown as blue crosses, reveal a systematic underestimation of the H_2_ mole fraction. This deviation remains within the uncertainty specified by the MFC manufacturer for lower H_2_ contents but slightly exceeds the uncertainty for mixtures with more than 50% H_2_. However, such high H_2_ concentrations were not reached in the actual isotope exchange experiment. The systematic nature of the deviation points to a potential inaccuracy in either the spectral transmission efficiency calibration of the spectrometer, the relative cross-section of the species, or the calibration of the MFCs. It should be noted that the MFCs were not factory-calibrated with the actual gases used. Instead, an internal database with gas-specific conversion factors was used. The manufacturer does not provide accuracy specifications for these conversion factors, and any inaccuracy therein could plausibly explain the trend observed in the data. The overall maximum deviation between the set H_2_ content and the measured value was 1.63%, confirming the overall accuracy of the mole fraction measurement to be better than 2%.

## Results and Discussion

Figure 4 shows the measured mole fractions of the detected species from the time D_2_ entered the cuvette up to 5500 s. The initial presence of approximately 0.19% N_2_ and 0.1% O_2_ is attributed to residual gases remaining after evacuation and minor leakage prior to sealing the cuvette. The elevated O_2_/N_2_ ratio (approximately 0.53 vs 0.27 in air) indicates a preferential retention of oxygen in the system, possibly due to its stronger interaction with the catalyst or cuvette surfaces during evacuation. Once the cuvette was sealed and the experiment began, there was no indication of ongoing leakage, as both the pressure and the N_2_ mole fraction remained constant throughout. The oxygen mole fraction, however, decreased by approximately one-third over the course of the experiment, suggesting an oxidation reaction involving either the catalyst surface or the hydrogen isotopologues, as discussed below. The primary reactant, D_2_, began to decrease immediately after time zero, reaching a final mole fraction of approximately 85%, corresponding to almost 15% conversion. The observed products of the scrambling reaction were H_2_ and HD. While the HD mole fraction increased steadily over time, H_2_ rose more rapidly at the beginning and then reached a plateau at approximately 2500 s. Quantitatively, HD reached a maximum mole fraction of 13.5%, while H_2_ peaked at 1.1%. These observations support the mechanism of primarily associative exchange of D_2_ molecules on the catalyst surface, as proposed by Limbach et al.^11^ The relatively low and quickly stabilizing H_2_ mole fraction argues against dominant dissociative exchange, which would typically produce higher H_2_ levels through surface atom equilibration. Compared to the ^1^H NMR data by Limbach et al.,^11^ the present measurements additionally allow for a quantitative determination of the HD-to-H_2_ ratio. Unraveling the processes occurring on the surface of the nanoparticles is beyond the scope of this study, as further diagnostic techniques are required. The gas-phase Raman data set will be used in a forthcoming paper focusing in detail on the kinetics and mechanisms, where it will be complemented by NMR data. The initial presence of 0.45% H_2_O in the cuvette, most likely originating from residual moisture in the catalyst that outgassed during evacuation, led to a secondary, superimposed isotopologue exchange reaction that is also observable in the data. H_2_O decreased rapidly, reaching the LOD, as defined in a later paragraph, at approximately 530 s. In contrast, D_2_O increased steadily after surpassing the LOD of approximately 177 ppm at 163 s. HDO exhibited a distinct maximum at 465 s with a mole fraction of approximately 1100 ppm before declining again and reaching approximately 420 ppm at 5500 s. This behavior suggests a stepwise isotopic exchange mechanism occurring on the catalyst surface. Initially, H_2_O from the gas phase binds to the catalyst surface, where one of its hydrogen atoms is replaced by a deuterium atom via surface-mediated exchange, forming HDO. Continued interaction with surface-bound deuterium leads to further substitution, converting HDO into D_2_O. A similar two-step mechanism has been proposed for aqueous-phase reactions on Pt nanoparticles by Leung et al.^35^ The overall exchange pathway can be summarized as

6 $$H_{2}O+D_{2}\to \mathrm{HDO}+\mathrm{HD}$$

7 $$\mathrm{HDO}+D_{2}\to D_{2}O+\mathrm{HD}$$

**Figure 4:**
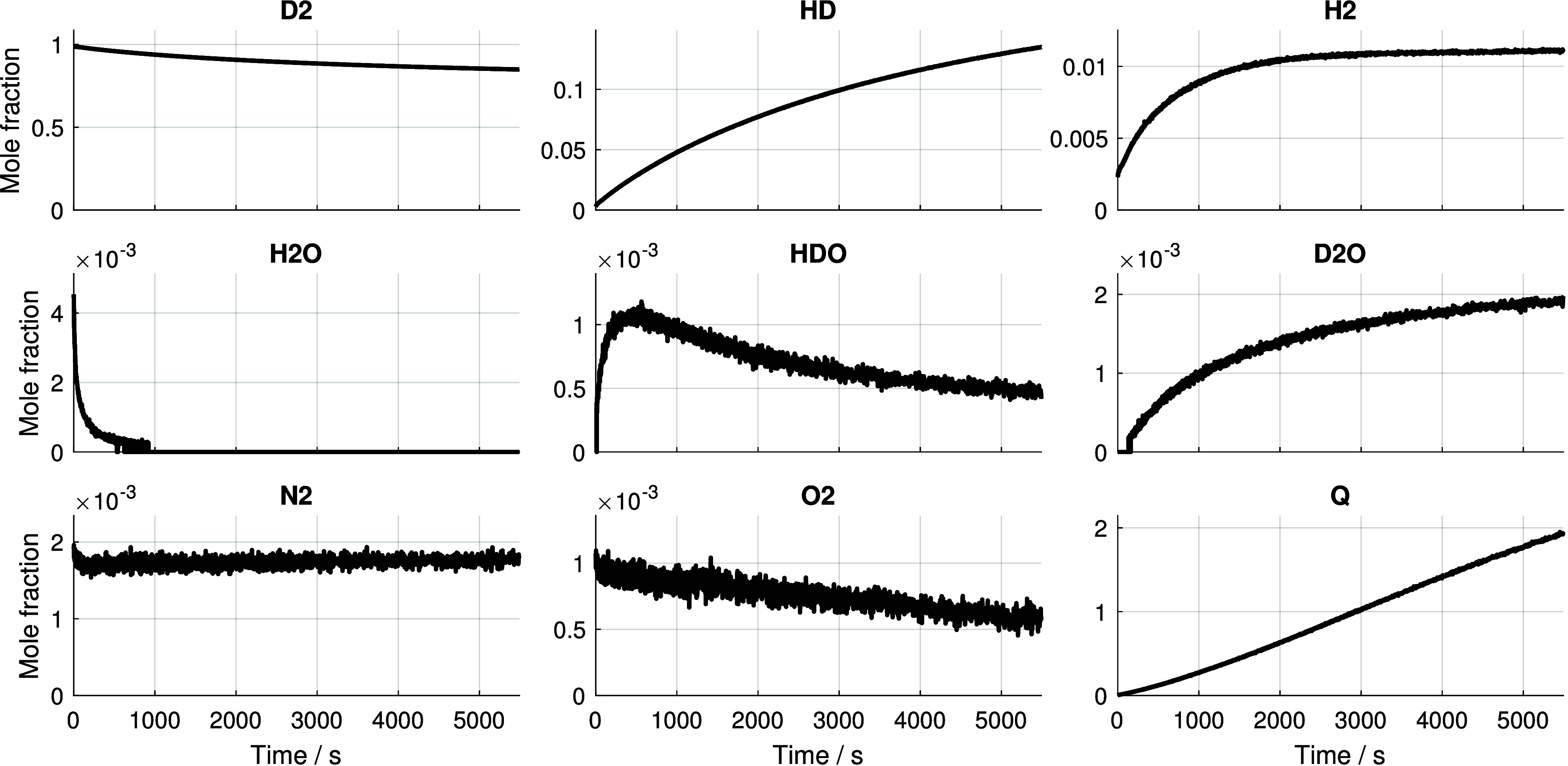
Time-resolved mole fractions of all detected species over the course of the isotope scrambling experiment at room temperature and 3 bar of pressure. The time-dependent reaction quotient *Q* for the hydrogen isotopologue reaction is plotted on the bottom right.

The sum of the mole fractions of all water isotopologues remained on average 0.2% throughout the experiment. Therefore, this superimposed exchange reaction is assumed to have a negligible influence on the hydrogen isotopologue exchange, which remains the main focus of this study. While the modest net increase in total water isotopologue content of approximately 0.04% absolute mole fraction from the disappearance of H_2_O to the end of the experiment cannot fully account for the observed decline in gas-phase O_2_, making bulk oxidation of D_2_ or HD unlikely, the decrease in O_2_ may still result from surface-mediated processes. Platinum nanoparticles are also employed as catalysts for oxidation reactions even at room temperature, including in systems similar to the one used here, where O_2_ is presumed to adsorb and react on the nanoparticle surface.^36^ Such sorption could plausibly explain the loss of gas-phase O_2_ without the formation of additional water isotopologues.

The time evolution of the reaction quotient *Q*(*t*) was monitored to track the progress of the isotope exchange reaction for the hydrogen isotopologues and is expected to converge toward the equilibrium constant *K*_*eq*_ over time. The reaction quotient *Q*(*t*) for the hydrogen isotopologues is calculated from the measured gas-phase mole fractions of HD, H_2_, and D_2_ as

8 $$Q\left(t\right)=\frac{x_{\mathrm{HD}}^{2}\left(t\right)}{x_{H_{2}}\left(t\right)\cdot x_{D_{2}}\left(t\right)}$$

At equilibrium and room temperature, this ratio is given by the equilibrium constant *K*_eq_ = 3.25. This value, calculated using isotope theory^37^ and confirmed experimentally,^38^ indicates that at equilibrium there is less HD in the gas phase than expected for a statistical distribution, where *K* would equal 4. The Raman data in Figure 4 clearly show that the reaction quotient did not reach the equilibrium constant within the measurement interval, as *Q*(*t*) continued to increase and reached a maximum value of 1.94. However, after the completion of the time-resolved measurements, the cuvette was kept sealed, and two additional measurements were performed at 51 and 115 h after the initial D_2_ introduction. These yielded *Q* values of 3.18 and 3.19, corresponding to relative deviations of 2.1% and 1.9% from the equilibrium constant, assuming the calculated *K*_eq_ value of 3.25 as ground truth. The close agreement of the late-time reaction quotient values with the theoretical equilibrium constant thus serves as additional validation for the accuracy and long-term stability of the mole fraction measurements, in line with the results presented in the Experimental Validation section.

The measured mole fraction of D_2_O is plotted over time in Figure 5, illustrating the emergence of D_2_O above the LOD. Between 100 and 130 s, no distinct D_2_O signal was identified, as the spectral contribution remained below the noise level. Although some D_2_O was likely present, its signal was too low to be detected. The detection limit is defined here as the point where the SNR, the ratio of the expected peak signal value in the library to the estimated noise, reaches 1. Between 130 and 160 s, the D_2_O signal fluctuated around the detection limit, resulting in alternating fits with and without a detectable D_2_O contribution. From 162 s onward, the signal was consistently above the noise level, and a nonzero mole fraction was retrieved. This time point corresponds to a mole fraction of 177 ppm and defines the LOD in this study. Compared to the initial D_2_ mole fraction of nearly 100%, this corresponds to a dynamic range of four orders of magnitude that can be captured by our system. The LOD of the system can be further improved by increasing the exposure time (at the expense of temporal resolution) or by raising the laser power by up to a factor of 10 with the current laser, although this would risk damaging the cuvette. Nevertheless, the LOD demonstrated here is, to the authors’ knowledge, unprecedented for nonintrusive kinetic investigations using in situ gas-phase Raman spectroscopy.

**Figure 5:**
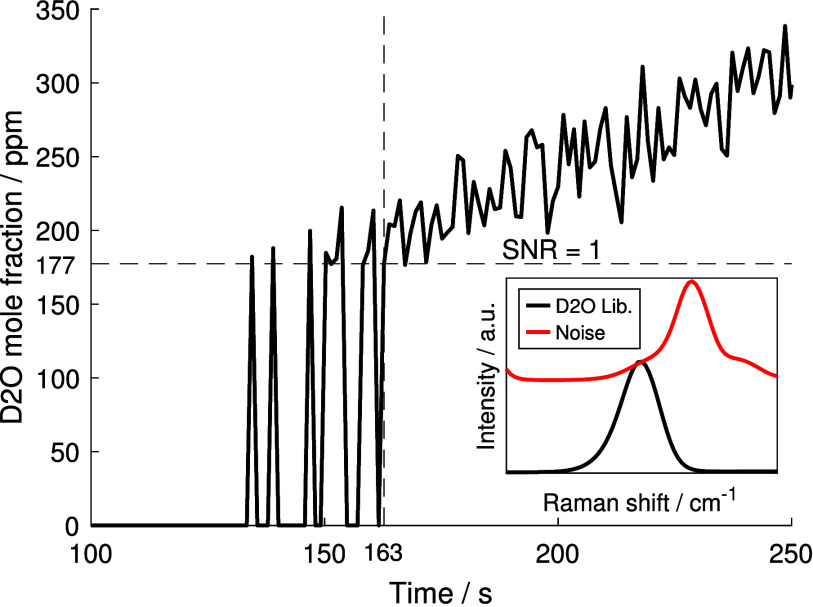
Temporal evolution of the D_2_O mole fraction in ppm. The transition from undetectable to confidently fitted signal lies at a peak SNR of 1 and defines the LOD, which is determined here to be approximately 177 ppm. The sub plot shows the fitted D_2_O library and the respective noise vector, dominated by the superimposed D_2_ signal, at the SNR = 1 threshold.

## Conclusion

This work demonstrates the unique potential of high-sensitivity gas-phase Raman spectroscopy using the DTRS for time-resolved in situ analysis of isotope exchange reactions over nanoparticle catalysts. Beyond confirming associative exchange as the dominant mechanism for hydrogen isotopologue scrambling, the system enabled simultaneous detection of minor species such as HDO and D_2_O in a closed sample reactor configuration with unprecedented sensitivity in the hundreds of ppm while maintaining a dynamic range of four orders of magnitude in mole fraction. The methodology combines a sophisticated spectral fitting routine with simulation-based species libraries, allowing for high accuracy and reliability even at low signal levels. The resulting quantitative species profiles provide a robust basis for kinetic modeling and mechanistic investigations, which will be addressed in a forthcoming study.

A key strength of the system is its adaptability: for slower reaction kinetics, the detection limit can be further improved by increasing the exposure time. Conversely, for faster processes, the exposure time can be decreased into the millisecond range to capture rapid dynamics at the cost of inferior detection limits. Additionally, the spatial resolution of the spectrometer and its thermography capabilities offer further potential for studying inhomogeneous and temperature-sensitive processes. This flexibility enables tailoring the measurement strategy to the specific reaction system under investigation.

Future applications may extend to a broader range of catalyst materials, including palladium, ruthenium, or even non-noble metals such as iron. The approach presented here provides a versatile and quantitative diagnostic tool for probing surface-mediated reactions in the gas phase, nonintrusively and in situ, opening new perspectives for kinetic studies in heterogeneous catalysis.

## Supplementary Material



## Data Availability

The time-resolved mole fraction data and associated uncertainty estimates generated in this study are publicly available through the TUdatalib repository at: 10.48328/tudatalib-1832.

## Notes

Detection and excitation scheme of the dual-track Raman spectrometeroptical components used in the spectrometer (PDF)
