# Supplementary material for: High-Sensitivity Gas-Phase Raman Spectroscopy for Time-Resolved In Situ Analysis of Isotope Scrambling over Platinum Nanocatalysts
Source: Anal Chem. 2025 Aug 13;97(33):18117–25. doi: 10.1021/acs.analchem.5c02840 (PMC12392255; doi:10.1021/acs.analchem.5c02840)
Supplement: Supplementary file 1 [file ac5c02840_si_001.pdf]

Supporting Information: High-Sensitivity Gas-Phase Raman Spectroscopy for Time-Resolved In-Situ Analysis of Isotope Scrambling over Platinum Nanocatalysts

K. Koschnick,[*,†,‡] A. M. Ferris,[¶,†] B. Zhang,[§] J. Lill,[‡,†] M. Stark,[‡,†,‖] A. Weinmann,[‖] H. H. Limbach,[⊥] T. Gutmann,[§] D. Geyer,[‡] and A. Dreizler[†]

†*Reactive Flows and Diagnostics, Department of Mechanical Engineering, Technical University of Darmstadt, Otto-Berndt-Straße 3, 64287 Darmstadt, Germany*

‡*Optical Diagnostics and Renewable Energies, Department of Mechanical and Plastics Engineering, University of Applied Sciences Darmstadt, Schöfferstraße 3, 64295 Darmstadt, Germany*

¶*Department of Mechanical and Aerospace Engineering, Princeton University, New Jersey 08544, USA*

§*Eduard-Zintl-Institute for Inorganic and Physical Chemistry, Technical University of Darmstadt, Alarich-Weiss-Straße 4, 64287 Darmstadt, Germany*

‖*Algorithms for Computer Vision, Imaging and Data Analysis, University of Applied Sciences Darmstadt, Schöfferstraße 3, 64295 Darmstadt, Germany*

⊥*Institute of Chemistry and Biochemistry, Freie Universität Berlin, Arminallee 22, 14195 Berlin, Germany*

E-mail: koschnick@rsm.tu-darmstadt.de

Additional Figure

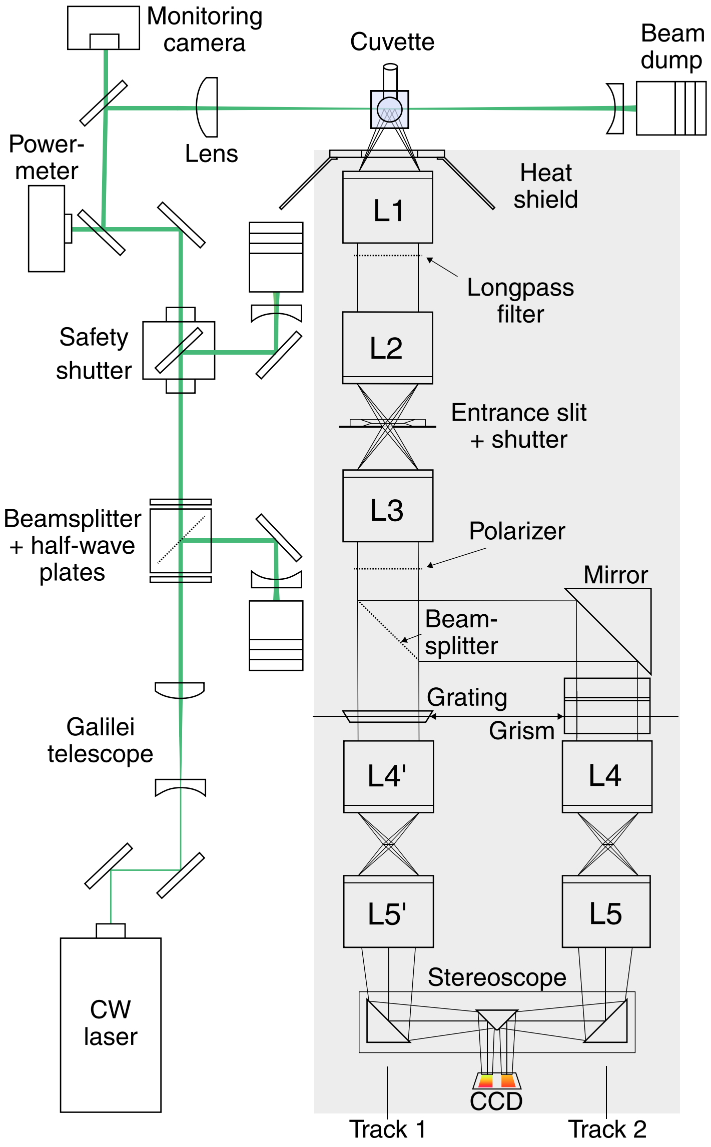

Figure S1: Detection scheme of the dual-track Raman spectrometer (gray shaded box) in combination with the excitation system and laser beam path (green) in top view. Track 2 is not used in this work.

Optical Components

Table S1: Optics used in the DTRS detection system

**Component**	**Manufacturer**	**Model / Spec**
L1	Pentax	67 Super-Takumar 105 mm f/2.4
L2	Zeiss	Planar T* 85 mm f/1.4 (F-Mount)
L3	Zeiss	Planar T* 85 mm f/1.4 (F-Mount)
L4 / L4'	Zeiss	Planar T* 85 mm f/1.4 (F-Mount)
L5 / L5'	Zeiss	Milvus 100 mm f/2.0 (Macro, F-Mount)
IR filter (heat shield)	Schott	KG2
Longpass filter	Semrock	BLP01-532R-50 (cut-on: 542 nm)
Beamsplitter	Edmund Optics	50/50 broadband non-polarizing
Polarizer	Moxtek	ProFlux ABGS5C
Mirrors	Edmund Optics	Dielectric broadband right-angle mirrors
Grating	Wasatch Photonics	Holographic transmission grating, 1064 l/mm
Grism	Wasatch Photonics	Holographic transmission grism, 3600 l/mm
